# Serum 25 hydroxyvitamin D levels and affecting factors among preconception fertile women

**DOI:** 10.1186/s12905-020-01018-1

**Published:** 2020-07-16

**Authors:** Haiyan Fan, Lingyun Hui, Xiaoting Yan, Wei Hou, E. Bai, Li Wang, Xuewen Yu

**Affiliations:** 1 Department of Obstetrics & Gynecology, First Affiliated Hospital of Xi’an Jiaotong University, Xi’an, 710061 China; 2 Department of General Medicine in Xi’an Jiaotong University, Xi’an, 710061 China; 3 Laboratory Department, First Affiliated Hospital of Xi’an Jiaotong University, Xi’an, 710061 China; 4 Department of Maternal Health Care, Maternal and Child Health Hospital of Shaanxi Province, Xi’an, 710002 China

**Keywords:** Preconception, Fertile women, 25 hydroxyvitamin D, Affecting factors

## Abstract

**Background:**

Recent study found that vitamin D before conception was considered as a potential additional determinant for achieving pregnancy and live births. The study aimed to evaluate the serum 25 hydroxyvitamin D (25(OH)D) levels and its affecting factors among preconception fertile women.

**Methods:**

This cross-sectional study enrolled 410 women aged 22–44 years who attended a preconception genetic counseling clinic from January 2018 to May 2019. Sociodemographic characteristics and reproductive history of women were collected, and height and weight were measured. Serum 25(OH)D concentration was assayed with chemiluminescence immunoassay. Descriptive statistics were used to examine serum 25(OH)D concentration, and socio-demographic characteristics and reproductive history among preconception women. Determinants of vitamin D deficiency and its affecting factors were assessed using χ2 test and logistic regression.

**Results:**

Findings showed 84.4% of women had serum 25(OH)D concentration below 20 ng/mL. Women working indoors as well as without a history of childbirth had significantly lower 25(OH)D levels compared with those non-working individuals and having delivered a previous child (both *P* < 0.05). The 25(OH)D levels were the lowest in winter among that in spring, summer, and autumn (all *P* < 0.001). Women in winter have significantly elevated OR of 5.00 (95%CI 1.75–14.25) to develop vitamin D deficiency. Seasonal variation in serum 25(OH)D levels was not present in non-working individuals and women aged 31–44 years.

**Conclusions:**

Vitamin D deficiency is common among preconception women especially nulliparous women and working women, which propose to screen serum 25(OH)D on preconception evaluation and emphasize need vitamin D supplements and get sunshine exposure.

## Background

Vitamin D3 is a lipid-soluble hormone that has well-established classical physiological function in maintaining calcium and phosphate homeostasis and promoting bone mineralization [1]. However, vitamin D3 is a pleiotropic signaling molecule, which plays numerous physiological roles ranging from regulating cellular proliferation and intracellular metabolism, and modulating innate and adaptive immune responses [2–4]. Indeed, current considerable amount of literature has been published on the association of vitamin D insufficiency with a number of diseases [5–10]. Especially, the inadequacy vitamin D during pregnancy is associated with adverse outcomes for mothers and the fetus or consequences on the offspring [11, 12]. Recently, a study showed that increased preconception vitamin D concentrations, not in early pregnancy, are associated with reduced pregnancy loss. Vitamin D before conception was considered as a potential additional determinant for achieving pregnancy and live births [13]. Moreover, childhood vitamin D insufficiency is strongly associated with maternal vitamin D insufficiency [14–17]. Women usually ignore vitamin D inadequacy because they do not feel unwell. Consequently, all the information and maternal/fetal health related issues associated with vitamin D inadequacy have highlighted the need to investigate the vitamin D level in fertile women who will become pregnant in following 3–6 months. So, the objective of study was to determine the levels of vitamin D and its association with possible determinant factors (age, body mass index, employment status, gravidity and parity, seasons) among fertile women who received preconception evaluation in Xi’an, China. We believe this information is valuable for women’s health care providers to provide better advice on vitamin D supplementation for women planning pregnancy as part of preconception counseling.

## Methods

### Research subjects and data collection

Fertile women who attended a preconception genetic counseling clinic in First Affiliated Hospital of Xi’an Jiaotong University in China were recruited in this cross-sectional study from January 2018 till May 2019. All the participant women aged 22–44 years received health counseling regarding preparing for a healthy pregnancy and infant at 3–6 months before they became pregnant with their new infant. The participants who were pregnant, complained muscular weakness and fatigue, diagnosed metabolic bone disease, and taking drugs affecting bone physiology, were excluded in the study. Data on the age, nationality, profession, gravidity, and parity of women were collected, followed by clinical examination (height and weight were measured, body mass index (BMI) was calculated) and laboratory results (serum 25-hydroxyvitamin D3 concentration). The study was approved by the Ethics Committee of the First Affiliated Hospital of Xi’an Jiaotong University, and written informed consent was provided from all participants.

March–May was constituted “spring”, June–August “summer”, September–November “autumn”, and December–February “winter” according to the climate in China. According to classification criteria of BMI in China, the BMI < 18.5 kg/m^2^ is as underweight, 18.5–23.9 kg/m^2^ normal weight, 24–27.9 kg/m^2^ overweight, ≥28 kg/m^2^ obese.

### Measurement of serum vitamin D

25-hydroxyvitamin D3 (25(OH)D) is the primary circulating form of vitamin D, which is the most abundant vitamin D metabolite and considered the best indicator of an individual’s vitamin D status within the human body [18]. Therefore, serum 25(OH)D was measured to determine vitamin D status with chemiluminescence immunoassay. Blood samples from fasting women were collected at times of usual blood draws. Serum 25(OH)D levels were detected using the “Elecsys 25(OH)D” kit (cobas® 25 Hydroxyvitamin D3 03314847 190, Roche) with a functional sensitivity of 4.0 ng/mL (to convert from ng/mL to nmol/L, multiply by 2.5) on a Roche Cobas E602 immunoanalyzer. All the assays were performed according to the manufacturer’s instruction. The interassay variability is 4.2–7.8% for 25(OH)D concentrations between13.8 and 42.5 ng/mL, based on a sample of 40 patients.

Vitamin D deficiency is defined as a serum 25(OH)D below 20 ng/mL (50 nmol/L), insufficiency as a 25(OH)D of 20–29 ng/mL (52–72.5 nmol/L), and an sufficient level as 30 ng/mL (75 nmol/L) or greater, according to the Institute of Medicine (IOM) and World Health Organization [19–21].

### Statistical analysis

Statistical analysis was performed using the SPSS-PC+ software (SPSS Inc., Chicago, IL, USA). Continuous variables were presented as mean ± standard deviations (SD) and categorical data as number (%). The Kolmogorov - Smirnov test was used to determine whether the parameters were normally distributed. The independent *t* test or ANOVA were used for normally distributed data of 25(OH)D levels. The Chi-Square test was used for categorical variables. Multiple liner regression analysis was performed with 25(OH)D level as a continuous variable and binary logistic regression model was performed with 25(OH)D level as a categorical with cut points at 20 ng/mL. A two-sided *p*-value ≤0.05 was considered statistically significant.

## Results

### General characteristics of participant women

A total of 410 fertile women between the ages 22 and 44 years attended the clinic at 3–6 months before they became pregnant were included in this study, of which 100% were Han nationality. The median BMI of them was 20.7 kg/m^2^ with a range of 19.5–23.0 kg/m^2^ (as 25–75%). Two hundred and forty (58.5%) women reported having one or more histories of pregnancy, and only 80 (19.5%) had delivered a previous child. One hundred and seventeen (28.5%) women underwent voluntary pregnancy termination. Three hundred and forty (82.9%) subjects were working women who were indoor job holders or office-based workers, while 70 (17.1%) were non-working individuals or housewives. A total of 128 (31.2%) serum samples were taken in spring, 102 (24.9%) in summer, 88 (21.5%) in autumn, and 92 (22.4%) in winter.

### The serum levels of 25(OH)D among preconceptional fertile women and factors associated with 25(OH)D level 

**Fig. 1:**
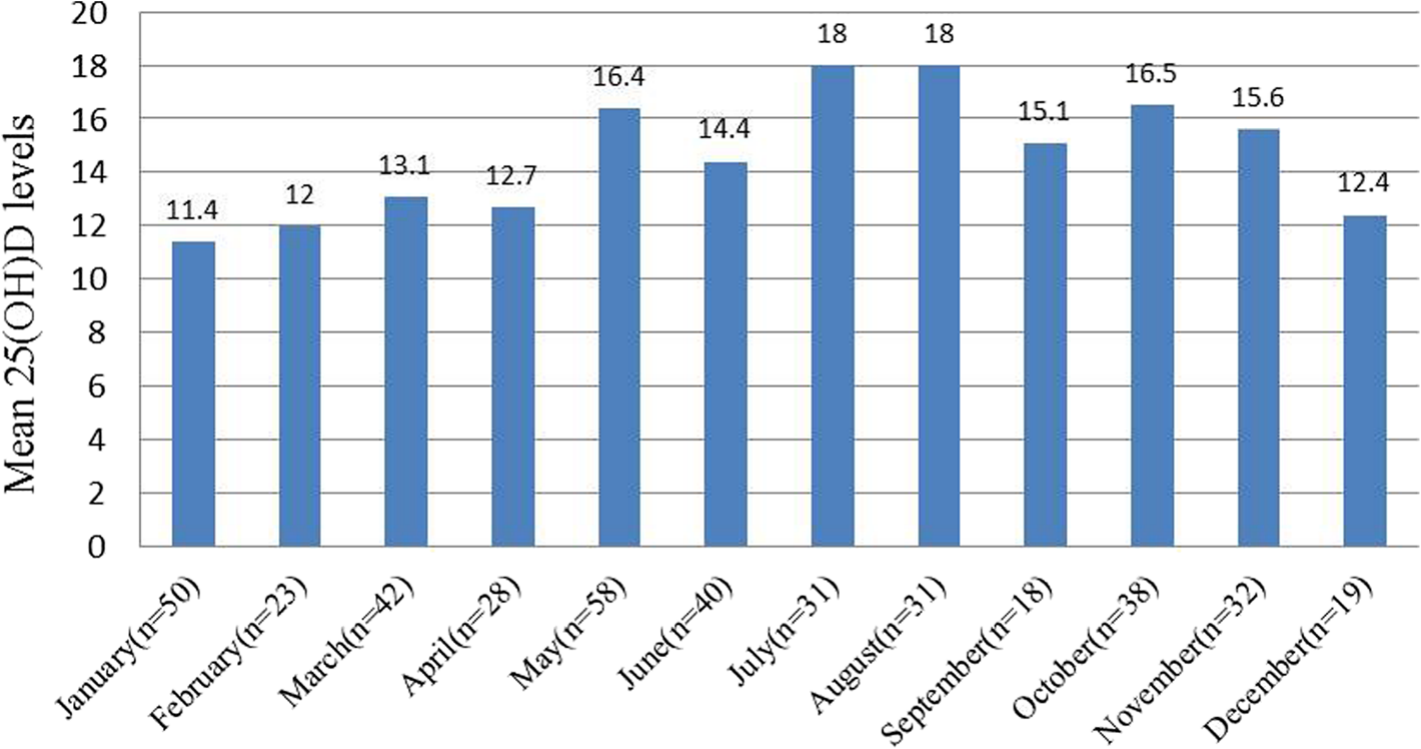
Mean serum 25(OH)D concentrations (ng/mL) based on month of serum collection among all participants

The mean serum 25(OH)D for the cohort was 14.7 ± 5.4 ng/mL. The concentrations ranged from a low of 4.5 ng/mL among young age women between 22 to 30 years to a high of 36.0 ng/mL among women aged 31 to 44 years. There was no effect of the gravidity, induced abortions, and BMI of the woman on the serum 25(OH)D level. There was however a statistically significant influence of the 25(OH)D level and season, number of deliveries, woman’s employment status, and age of women (All *p*  <  0.05). Table 1 shows the mean of 25(OH)D levels for specified subgroups. Women aged 22–30 years had significantly lower 25(OH)D level compared with those between 31 and 44 years (14.3 ± 5.0 ng/mL vs 15.6 ± 6.0 ng/mL, *P* < 0.05). The mean vitamin D level was significantly lower in working women compared to non-working individuals or housewives (14.3 ± 5.2 ng/mL vs 16.5 ± 5.8 ng/mL, *P* < 0.01). Women who had delivered a previous child had higher 25(OH)D levels in comparison to women without a history of childbirth (15.9 ± 5.7 ng/mL vs 14.4 ± 5.3 ng/mL, *P* < 0.05). Mean serum 25(OH)D concentrations varied by month and season of sample collection. The 25(OH)D concentrations were the lowest in winter among that in spring, summer, and autumn (all *P* < 0.001). The range of 25(OH)D concentration was from 18.0 ng/mL in July and August to 11.4 ng/mL in January (Fig. 1).

**Table 1:** Association of serum 25(OH)D level with woman's general characteristics and season

Characteristics	Group (n)	Serum 25(OH)D (mean ± SD ng/mL)	*t/F*	*p*
Age	22–30 years (277)	14.3 ± 5.0	2.203	0.029
	31–44 years (133)	15.6 ± 6.0		
BMI (kg/m^2^)	Underweight (46)	13.7 ± 4.5	1.273	0.281
	Normal (293)	14.9 ± 5.4		
	Overweight and Obese (71)	14.4 ± 5.7		
Gravidity	No (170)	14.1 ± 5.1	1.813	0.071
	One and more (240)	15.1 ± 5.6		
Parity	No (330)	14.4 ± 5.3	2.290	0.023
	One (80)	15.9 ± 5.7		
Numbers of induced abortion	No (293)	14.7 ± 5.7	0.143	0.866
	One (85)	14.7 ± 5.2		
	Two and more (32)	15.2 ± 6.2		
Employment status	Working (340)	14.3 ± 5.2	3.127	0.002
	Non-working (70)	16.5 ± 5.8		
Season	Spring (128)	14.5 ± 5.1^#^	16.417	0.000
	Summer (102)	16.6 ± 5.0		
	Autumn (88)	15.9 ± 5.6		
	Winter (92)	11.8 ± 4.8*		

*Significantly lower compared to spring, summer, and autumn (all *p* < 0.01)

^#^Significantly lower compared to summer (*p* < 0.01)

For the stepwise multiple linear regression analysis (Table 2), the three variables, season, woman’s employment status, and parity are correlated to concentrations of serum 25(OH)D of the women [*R*^*2*^ = 0.113, *F* = 17.306, *p* = 0.000]. The winter, working women as well as nulliparous women contributed to low serum 25(OH)D concentrations among the women. Furthermore, woman’ age and BMI was no longer a significant predictor of vitamin D after adjusting for all other factors.

**Table 2:** Determinants of 25(OH)D level from a stepwise multiple linear regression

	Unstandardized coefficients		*p*
	B	S.E	
Constant	12.306	0.900	0.000
Season	1.472	0.238	0.000
Employment	−1.789	0.688	0.010
Parity	1.390	0.655	0.034

Further, the mean serum 25(OH)D concentration differed by season after stratification by age and by women’s employment status. Seasonal variation of 25(OH)D levels was present in working individuals (F = 17.013, *P* = 0.000), with lower concentrations in winter (11.5 ± 4.6) and spring (13.7 ± 4.6) than in summer (16.4 ± 4.9) and autumn (15.9 ± 5.6) (Table 3). This seasonal variation was quantitatively less among women aged 31–44 years when compared with young women aged 22–30 years. For example, the difference between winter and summer mean 25(OH)D levels was 5.1 ng/mL (95% CI: 3.5–6.7 ng/mL) among young women aged 22–30 years, and 3.8 ng/mL (95% CI: 0.9–6.8 ng/mL) among women aged 31–44 years.

**Table 3:** The mean 25(OH)D levels by season after stratification by age and by women’s employment status (mean ± SD ng/mL)

Season	Employment status		Age	
	Working (*n* = 285)	Non-working (*n* = 57)	22–30 years (*n* = 241)	31–44 years (*n* = 101)
Spring	13.7 ± 4.6	17.3 ± 5.8	14.5 ± 5.0	14.7 ± 5.5
Summer	16.4 ± 4.9	17.7 ± 5.5	16.1 ± 4.9	17.5 ± 5.1
Autumn	15.9 ± 5.6	15.6 ± 5.7	15.7 ± 5.1	16.3 ± 6.7
Winter	11.5 ± 4.6	13.8 ± 6.0	11.0 ± 3.8	13.6 ± 6.5
*F*	17.013	1.384	15.655	2.664
*p*	0.000	0.256	0.000	0.051

### Vitamin D deficiency in seasons and in terms of maternal variables

A large proportion (346,84.4%) of women had serum 25(OH)D concentration below 20 ng/mL, indicating that they were deficient in vitamin D. Only 3 (0.7%) women were sufficient with serum 25(OH)D (30 ng/mL or more), 61 (14.9%) were insufficient (20–30 ng/mL). Consistent with the mean 25(OH)D data, 295 (86.8%) working women were vitamin D deficient in contrast to 51 (72.9%) non-working individuals or housewives (χ2 = 8.523, *p* = 0.004). In line with seasonal variation of vitamin D levels, the percentage of women with 25(OH)D concentrations below 20 ng/mL was significantly higher in winter (*n* = 87, 94.6%) than in spring (*n* = 110, 85.9%), summer (*n* = 80, 78.4%), and autumn (*n* = 69,78.4%) (χ2 = 12.602, *p* = 0.006). A higher proportion (*n* = 286, 86.7%) of nulliparous women with 25(OH)D < 20 ng/mL was found compared to women (*n* = 60, 75%) who had delivered a previous child (χ2 = 6.653, *p* = 0.010).

Binary logistic regression analysis was performed after that 25(OH)D was specified as categorical with cut point at 20 ng/mL (deficient < 20 ng/mL, non-deficient≧20 ng/mL). The results establish that women in winter have significantly elevated OR of 5.00 to develop vitamin D deficiency, although, it is important to note the wide confidence interval (95%CI 1.75–14.25). The nulliparous women have 2.32 higher odds of deficiency than women who had delivered a previous child (95% CI 1.08–3.98). The non-working women or housewives have a significantly lower susceptibility to develop vitamin D deficiency than working women (OR 0.49, 95% CI 0.26 to 0.96) (Table 4). This is consistent with the results of the multiple linear regression analysis described above.

**Table 4:** Results from binary logistic regression analysis for vitamin D deficiency

	Subcategory	OR	p	95% Confidence interval
Season			0.007	
	winter	5.000	0.003	1.754–14.250
	Spring	1.925	0.080	0.925–4.005
	Summer	1.014	0.970	0.500–2.055
	Autumn	1.00		
Employment tatus	Non-working	0.494	0.036	0.256–0.955
	Working	1.00		
Parity	Nulliparous	2.320	0.030	1.076–3.981
	One	1.00		

## Discussion

There are limited data about vitamin D deficiency in healthy nonpregnant fertile women who are planning to get pregnant. This study was focused on comparative affluent and healthy fertile women to receive preconception evaluation but their vitamin D levels were found to be low. Serum 25(OH)D levels reflect body stores of vitamin D [1]. Nearly 84 % of fertile nonpregnant women were 25(OH)D deficient in this study. Especially in young women aged 22–30 years, serum 25(OH)D level was lower in comparison with women between 31 and 44 years. These findings were nearly consistent with a population-based study by Fang et al. [22] reported that serum 25(OH)D level was the lowest in the 18–29-year-old group (46.66 ± 12.98 nmol/L) among all participant women aged 18–49 years in Tianjin, China. Our findings are also supported by a recent study from Pakistanon vitamin D deficiency in asymptomatic healthy young students. Almost 89 % of participants were vitamin D deficient (25(OH)D < 20 ng/mL) [23].

This is alarming as those women are planning to get pregnant because routine pre-conception check-up projects (Women receive routine pre-pregnancy check-up at 3–6 months before they became pregnant in China) do not include measurement of serum 25(OH)D. Several studies have described an association between insufficiency or deficiency in 25(OH)D levels and adverse pregnancy outcomes including gestational diabetes mellitus, preeclampsia, preterm delivery, recurrent abortion, and intrauterine growth restriction [24–26]. More recent data showed that preconception vitamin D status was associated with male live birth [27]. Increased pre-conception vitamin D concentrations are associated with reduced pregnancy loss [13].

In this study, we also found that working women displayed a lower level of vitamin D relative to non-working individuals. Sowah, et al. also observed a higher prevalence of vitamin D deficiency in all occupational populations examined than the reported population burden of vitamin D deficiency in multiple populations, suggesting that workers may be particularly vulnerable to vitamin D deficiency [28]. Among the occupations of those women participating in this study, those women almost were office-based workers, who spend a considerable amount of time indoors without sunshine exposure, had a greater risk of vitamin D deficiency compared to women who were non-working. Women in an indoor setting would be expected to get their sunshine exposure during mornings and evenings, when sunlight intensity is relatively low. As we all know, vitamin D3 is needed from not only adequate intake from diet or supplements but also biosynthesis in the skin in response to sufficient exposure to sunlight. Several studies have concerned seasonal variation in serum 25(OH)D concentrations [29–31]. This study also found that serum 25(OH)D concentration was the lowest in January and the highest in July and August. Almost 94 % of healthy preconception fertile women had serum 25(OH)D levels < 20 ng/mL during winter in Xi’an, China. Similar to above results, traveling to a warmer climate during winter/spring was associated with higher serum 25(OH)D according to a prevalence of vitamin D deficiency in healthy women of reproductive age in Canada [32]. Statistics Canada found that sunlight exposure more than 1 h per day and month of blood collection were positively associated with higher serum 25OHD levels [33].

It is interesting that when analyzed by season, non-working individuals and women between 31 and 44 years did not have significant winter decline in serum 25(OH)D levels seen in working individuals and young women aged 22–30 years. This altered pattern may be due to trends of increased multivitamin supplements among women aged 31–44 years. Mitchell et al. study suggests that increased oral intake of vitamin D compensated for decreased ultraviolet radiation B exposure [34]. There may also have been differences in sun vacation or outdoor time with no sun protection (i.e. no protection from the sun using clothing or sunscreen) between working and non-working women.

In contrast to many studies [35, 36], we found no association between BMI and serum 25(OH)D, which could be accounted for the normal and low BMI of our participates. Indeed, about 84% of women had BMI < 24 kg/m^2^, only one had BMI > 28 kg/m^2^ indicating obese.

Our study had some limitations. We did not collect information regarding dietary vitamin D intake, vitamin D/multivitamin supplements, or measures of plasma parathyroid hormone. Regarding occupation, we do not have data on natural ultraviolet B exposure time or amount, which may confound the effects of ultraviolet B in different subgroups. Finally, our data are cross-sectional, thus, we do not have insight into longitudinal changes in 25(OH)D, and we do not provide reproductive outcomes.

## Conclusions

Overall, we found a large proportion of vitamin D deficiency in this “lower-risk” cohort. Women working in an institutional setting, young women and living in winter are more prone to vitamin D deficiency due to reduced solar exposure. Decreased serum vitamin D levels among preconception fertile women may predispose to increased risk for adverse pregnancy [13, 26]. Therefore, we propose that healthy fertile women who are pregnancy in following 3–6 months may be screened for serum 25(OH)D levels on preconception valuation and emphasize need vitamin D supplements and get sunshine exposure.

## Data Availability

The data used during the current study are available from the corresponding author on reasonable request.

## Abbreviations

25(OH)D: 25-hydroxyvitamin D3
BMI: Body mass index
95%CI: 95% confidence interval
OR: Odds ratio

## References

[1] Holick MF (2007). Vitamin D deficiency. N Engl J Med.

[2] Caprio M, Infante M, Calanchini M, Mammi C, Fabbri A (2017). Vitamin D: not just the bone. Evidence for beneficial pleiotropic extraskeletal effects. Eat Weight Disord.

[3] Bikle DD (2011). Vitamin D regulation of immune function. Vitam Horm.

[4] Lagishetty V, Liu NQ, Hewison M (2011). Vitamin D metabolism and innate immunity. Mol Cell Endocrinol.

[5] Muris A-H, Rolf L, Broen K, Hupperts R, Damoiseaux J, Smolders J (2016). A low vitamin D status at diagnosis is associated with an early conversion to secondary progressive multiple sclerosis. J Steroid Biochem Mol Biol.

[6] Marquina C, Mousa A, Scragg R, de Courten B (2019). Vitamin D and cardiometabolic disorders: a review of current evidence, genetic determinants and pathomechanisms. Obes Rev.

[7] Sassi F, Tamone C, D' Amelio P. Vitamin D: nutrient, hormone, and immunomodulator. Nutrients. 2018; 10(11). pii: E1656. 10.3390/nu10111656 PMC6266123 30400332

[8] Zheng JS, Imamura F, Sharp SJ, van der Schouw YT, Sluijs I, Gundersen TE, Ardanaz E, Boeing H, Bonet C, Gómez JH (2019). Association of plasma vitamin D metabolites with incident type 2 diabetes: EPIC-inter act case-cohort study. J Clin Endocrinol Metab.

[9] Mondul AM, Weinstein SJ, Layne TM, Albanes D (2017). Vitamin D and cancer risk and mortality: state of the science, gaps, and challenges. Epidemiol Rev.

[10] Autier P, Boniol M, Pizot C, Mullie P (2014). Vitamin D status and ill health: a systematic review. Lancet Diabetes Endocrinol.

[11] Kiely ME, Zhang JY, Kinsella M, Khashan AS, Kenny LC (2016). Vitamin D status is associated with uteroplacental dysfunction indicated by pre-eclampsia and small-for-gestational-age birth in a large prospective pregnancy cohort in Ireland with low vitamin D status. Am J Clin Nutr.

[12] Bärebring L, Bullarbo M, Glantz A, Hulthén L, Ellis J, Jagner Å, Schoenmakers I, Winkvist A, Augustin H (2018). Trajectory of vitamin D status during pregnancy in relation to neonatal birth size and fetal survival: a prospective cohort study. BMC Pregnancy Childbirth.

[13] Mumford SL, Garbose RA, Kim K, Kissell K, Kuhr DL, Omosigho UR, Perkins NJ, Galai N, Silver RM, Sjaarda LA (2018). Association of preconception serum 25-hydroxyvitamin D concentrations with livebirth and pregnancy loss: a prospective cohort study. Lancet Diabetes Endocrinol.

[14] Dijkstra SH, van Beek A, Janssen JW, de Vleeschouwer LH, Huysman WA, van den Akker EL (2007). High prevalence of vitamin D deficiency in newborn infants of high-risk mothers. Arch Dis Child.

[15] Özdemir AA, Gündemir YE, Küçük M, Sarıcı DY, Elgörmüş Y, Çağ Y, Bilek G (2018). Vitamin D deficiency in pregnant women and their infants. J Clin Res Pediatr Endocrinol.

[16] Dawodu A, Wagner CL (2012). Prevention of vitamin D deficiency in mothers and infants worldwide - a paradigm shift. Paediatr Int Child Health.

[17] Ariyawatkul K, Lersbuasin P (2018). Prevalence of vitamin D deficiency in cord blood of newborns and the association with maternal vitamin D status. Eur J Pediatr.

[18] Rosen CJ (2011). Clinical practice. Vitamin D insufficiency. N Engl J Med.

[19] Esmaeili SA, Mohammadian S, Radbakhsh S, Momtazi-Borojeni AA, Parizi KP (2019). AtabatiHadi, MardaniF, Saburi E, Moghaddam AS. Evaluation of vitamin D3 deficiency: a population-based study in northeastern Iran. J Cell Biochem.

[20] Holick MF, Binkley NC, Bischoff-Ferrari HA, Gordon CM, Hanley DA, Heaney RP, Murad MH (2011). Weaver CM; Endocrin society. Evaluation, treatment, and prevention of vitamin D deficiency: an Endocrine Society clinical practice guideline. J Clin Endocrinol Metab.

[21] Spiro A, Buttriss JL. Vitamin D: An overview of vitamin D status and intake in Europe. Nutr Bull 2014; 39(4): 322–350. 10.1111/nbu.12108 PMC4288313 25635171

[22] Fang F, Wei H, Wang K, Tan L, Zhang W, Ding L, Liu T, Shan Z, Zhu M (2018). High prevalence of vitamin D deficiency and influencing factors among urban and rural residents in Tianjin, China. Arch Osteoporos.

[23] Nadeem S, Munim TF, Hussain HF, Hussain DF (2018). Determinants of vitamin D deficiency in asymptomatic healthy young medical students. Pak J Med Sci.

[24] Heyden EL, Wimalawansa SJ (2018). Vitamin D: effects on human reproduction, pregnancy, and fetal well-being. J Steroid Biochem Mol Biol.

[25] Olmos-Ortiz A, Avila E, Durand-Carbajal M, Díaz L (2015). Regulation of calcitriol biosynthesis and activity: focus on gestational vitamin D deficiency and adverse pregnancy outcomes. Nutrients.

[26] Wilson RL, Leviton AJ, Leemaqz SY, Anderson PH, Grieger JA, Grzeskowiak LE, Verburg PE, McCowan L, Dekker GA, Bianco-Miotto T (2018). Vitamin D levels in an Australian and New Zealand cohort and the association with pregnancy outcome. BMC Pregnancy Childbirth.

[27] Purdue-Smithe A, Kim K, Nobles C, Schisterman E, Schliep K, Perkins N, Sjaarda L, Freeman J, Robinson S, Radoc J, et al. Preconception vitamin D status and offspring sex ratio among women with prior pregnancy loss (OR17–05-19). Curr Dev Nutr. 3(Suppl 1).2019; pii: nzz039.OR17–05-19.

[28] Sowah D, Fan X, Dennett L, Hagtvedt R, Straube S (2017). Vitamin D levels and deficiency with different occupations: a systematic review. BMC Public Health.

[29] Hansen L, Tjønneland A, Køster B, Brot C, Andersen R, Cohen AS, Frederiksen K, Olsen A (2018). Vitamin D status and seasonal variation among Danish children and adults: a descriptive study. Nutrients..

[30] Niculescu DA, Capatina CAM, Dusceac R, Caragheorgheopol A, Ghemigian A, Poiana C (2017). Seasonal variation of serum vitamin D levels in Romania. Arch Osteoporos.

[31] Serdar MA, Batu Can B, Kilercik M, Durer ZA, Aksungar FB, Serteser M, Coskun A, Ozpinar A, Unsal I (2017). Analysis of changes in parathyroid hormone and 25 (OH) vitamin D levels with respect to age, gender and season: a data mining study. J Med Biochem.

[32] Gagnon C, Baillargeon J-P, Desmarais G, Fink GD (2010). Prevalence and predictors of vitamin D insufficiency in women of reproductive age living in northern latitude. Euro J Endocrinol.

[33] Langlois K, Greene-Finestone L, Little J, Hidiroglou N, Whiting S (2010). Vitamin D status of Canadians as measured in the 2007 to 2009 Canadian health measures survey. Health Rep.

[34] Mitchell DM, Henao MP, Finkelstein JS, Burnett-Bowie SA (2012). Prevalence and predictors of vitamin D deficiency in healthy adults. Endocr Pract.

[35] Ho V, Danieli C, Abrahamowicz M, Belanger A-S, Brunetti V, Delvin E, Lacaille J, Koushik A (2018). Predicting serum vitamin D concentrations based on self-reported lifestyle factors and personal attributes. Bri J Nutr.

[36] Manoy P, Yuktanandana P, Tanavalee A, Anomasiri W, Ngarmukos S, Tanpowpong T, Honsawek S (2017). Vitamin D supplementation improves quality of life and physical performance in osteoarthritis patients. Nutrients..

